# Precise and simultaneous SERS detection of sertraline and serotonin on large-scale sub-20 nm plasmonic gold nanocone arrays

**DOI:** 10.1007/s00604-026-08164-7

**Published:** 2026-06-06

**Authors:** Klára Gajdošová, Thanh-Lam Bui, Zuzana Chaloupková, S. M. Hossein Hejazi, Radek Zbořil, Štěpán Kment, Václav Ranc, Kateřina Poláková

**Affiliations:** 1 Regional Centre of Advanced Technologies and Materials, Czech Advanced Technology and Research Institute (CATRIN), Palacký University Olomouc, Šlechtitelů 27, Olomouc, 783 71 Czech Republic; 2 Department of Physical Chemistry, Faculty of Science, Palacký University Olomouc, 17 Listopadu 12, Olomouc, 771 46 Czech Republic; 3 Nanotechnology Centre, Centre for Energy and Environmental Technologies, VSB – Technical University of Ostrava, 17. listopadu 15, Poruba, Ostrava, 708 00 Czech Republic; 4 Institute of Molecular and Translational Medicine, Faculty of Medicine and Dentistry, Palacký University and Faculty Hospital Olomouc, Hněvotínská 5, Olomouc, 775 15 Czech Republic

**Keywords:** Surface-enhanced Raman spectroscopy (SERS), Anodic aluminum oxide (AAO), Gold nanocone, Antidepressant, Sertraline, Serotonin

## Abstract

A highly sensitive and reproducible SERS platform for sertraline and serotonin detection is presented using large-scale, cost-effective, and facile fabrication of sub-20 nm nanogaps in plasmonic gold nanocone (AuNC) arrays. They , ensure a high density of uniform electromagnetic enhancement sites, giving the platform “hot-spot” consistency. The sertraline limit of detection (LOD) of 88 ng/mL is two orders of magnitude lower than clinical values, representing the lowest SERS-based LOD for sertraline reported to date. The LOD for serotonin, 27 ng/mL, is lower than its blood levels, even in depression. Moreover, simultaneous, parallel detection of both analytes within artificial cerebrospinal fluid (aCSF) mimicking physiological composition was achieved with no sample pretreatment, highlighting the platform’s high selectivity. Our method shows great potential for complex antidepressant therapeutic monitoring in clinical conditions.

**Graphical abstract:**

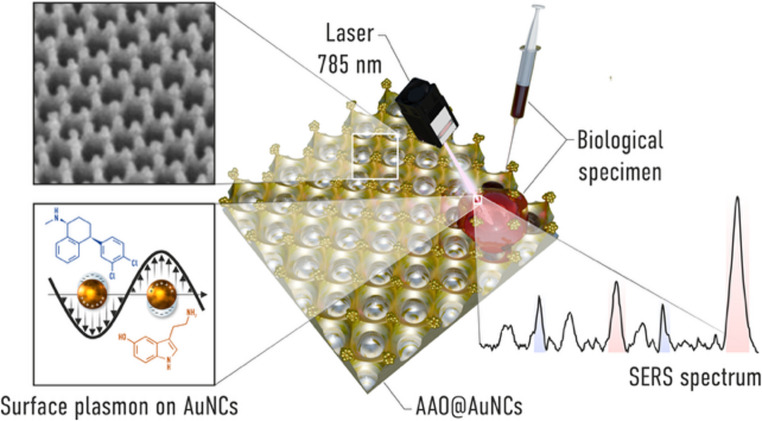

**Supplementary Information:**

The online version contains supplementary material available at 10.1007/s00604-026-08164-7.

## Introduction

Over 40 million people in Europe alone and around 5% of the overall human population suffer from depression, according to the World Health Organization (WHO) [1]. The disease is, in many cases, treated by administering sertraline as a preferred medication strategy over other on-the-market antidepressants for its higher efficacy and tolerability [2]. However, the current treatment is often suboptimal, which can be partially explained by the genetic polymorphisms of cytochromes CYP2B6 and CYP2C19 [3]. To address the therapeutic drug monitoring (TDM) of sertraline, multiple analytical methods were developed and evaluated. A machine learning-based approach was suggested to evaluate its pharmacokinetics in children and adolescents, aiming at predicting side effects in this sensitive age group, with satisfactory results [4]. Sertraline is typically detected by high-performance liquid chromatography [5], followed by optical [6, 7] or mass spectrometric [8] detection. The most typical matrix is blood, with limits of detection ranging from tens to units of ng/mL [3, 5, 6]. The limit of detection from post-mortem cerebrospinal liquor is 40 ng/mL [6]. For the case of TDM of sertraline, microsampling using the hemaPEN [8] or volumetric absorptive microsampling (VAMS) [7] approach has been suggested; both approaches are favorable for at-home self-sampling. The detection was performed with liquid chromatography – mass spectroscopy (LC-MS/MS) or high-performance liquid chromatography – spectrophotometric and spectrofluorimetric detection (HPLC-UV-FL), respectively, reaching a limit of quantification of 5 ng/mL in both cases [7, 8].

Serotonin (5-hydroxytryptamine; 5-HT) is a monoamine neurotransmitter that acts as a neuromodulator in the central nervous system, and its levels have been previously linked to depression, particularly through its interactions with 5-HT1, 5-HT2, and 5-HT4 receptors. Although the recent understanding is that serotonin concentration levels are not directly linked to the evolution of depression, their monitoring provides valuable information during the progression of treatment [9]. Several approaches aiming at the analysis of serotonin in clinical samples are based on high-performance liquid chromatography – electrochemical detection (HPLC-ECD) [10] or LC-MS [11] with the LOD reaching below units of ng/mL. However, all of these above-mentioned methods, although sophisticated and capable of ultra-low LODs, are time-consuming and expensive, and often require sample pre-treatment.

Interestingly, methods based on molecular spectroscopy offer promising alternatives to separation techniques. Surface-enhanced Raman spectroscopy (SERS) is a rapid method that is exceptionally suitable for analyzing bioanalytes [12]. SERS is non-destructive, minimally invasive, and requires small sample volumes, making it suitable for point-of-care and ex vivo applications [12, 13]. Multivariate interpretation of the spectra enables the simultaneous analysis of multiple components at physiological concentrations [14, 15]. Numerous SERS-based detection of serotonin have been explored, using predominantly dispersions of gold or silver nanoparticles (NPs), whereas SERS detection of sertraline was reported only in higher concentrations, using SiO_2_@Ag nanocluster modified with serotonin transporter or nanogaps of silver octahedral hollow cages at concentration 2 and 1 mg/mL, respectively [16, 17]. The SERS detection of serotonin using dispersions of plasmonic nanoparticles is often complex, e.g., two types of AuNPs functionalized with different selectors that bind to functional groups of serotonin [18], or a sandwich-type structure of two types of Au-based hybrids, which both require two hours of incubation with the analyte [19]. As indicated by these examples, dispersions can possess multiple drawbacks, including random aggregation, a limited timescale for detection, disposability, poor long-term stability, and reproducibility. This could be addressed by attaching plasmonic nanoparticles to a solid substrate. One type of SERS substrate used for detecting serotonin consists of a functionalized glass, such as a glass slide with electrodeposited AuNPs; however, the spectra of serotonin are not reproducible, even at high concentrations (180 µg/mL) [20]. Better spectral quality was achieved with optical fiber functionalized with AuNPs, achieving LOD 1.8 µg/mL [21], or a microscope glass functionalized with AuNPs, capable of nanomolar detection [22]. A modified filter paper with immobilized AuNPs in the shape of concave cubes enabled the detection of serotonin at a concentration of 18 ng/mL [23]. AgNPs embedded in porous silicon template achieved an exceptional LOD of 0.1 picomolar [24]. These results highlight the advantages of SERS substrates with a uniform array of plasmonic nanoparticles over plasmonic dispersions. A large-scale synthesis of such materials, derived from inexpensive precursors, is therefore a key research focus. Since the discovery of nanoporous anodic aluminum oxide (AAO) material by Masuda and Fukuda, tremendous efforts have been made to utilize the AAO template for various applications, including drug delivery, nanophotonics, or photocatalysis [25–28]. SERS substrates based on AAO were prepared via arraying noble metallic nanomaterials onto the AAO template by taking advantage of the uniformity in nanopore diameter, interpore distance, and wall thickness of the AAO substrate. The relative standard deviation of analysis performed using AAO-based Au SERS substrates (within one batch) usually reaches below 10%, when tested on reporter molecules [29–32]. This work presents a lage-scale synthesis of gold nanocones array on top of morphologically interesting sharp-tip AAO template. The substrate presents with exceptional uniformity, reproducibility and stability, vital parameters for point-of-care use.

Analysis of sole sertraline levels may not be clinically sufficient to provide a complex image of its therapeutic impact. Parallel analysis of serotonin levels could provide beneficial information, offering a clearer picture. In this work, we demonstrate the first SERS-based biosensing approach for the multiplexed analysis of serotonin and sertraline. The exceptionally homogeneous, large-scale array of sub-20 nm gold nanocones (AuNCs) fabricated on the sharp-tip AAO substrate was used as a reliable signal-enhancing platform. It presents a novel SERS-based approach for detecting sertraline in sub-physiological concentrations. The limits of detection reached 27 ng/mL for serotonin and 88 ng/mL for sertraline, respectively. To test the method’s performance and applicability, parallel SERS analysis of low concentrations of serotonin and sertraline in artificial cerebrospinal fluid spiked with physiological levels of interferents was performed, demonstrating its potential clinical applicability, particularly in therapeutic drug monitoring.

## Materials and methods

### Chemicals

Aluminum foils (Thermo Fisher Scientific, 99.99%, 0.25 mm thickness), oxalic acid (Sigma, 99%), perchloric acid (Sigma, 70%), phosphoric acid (Sigma, 85%), chromium (VI) oxide (Sigma, ≥ 99%), gold coated silicon wafer (Sigma Aldrich, 99.999% Au, layer thickness 1000 Å), gold disc (Kurt J. Lesker, 99.99%), CY^TM^3 Mono NHS Ester (Sigma Aldrich, 100%), sertraline hydrochloride (Sigma Aldrich, pharmaceutical secondary standard; certified reference material, 100%), serotonin hydrochloride (Sigma Aldrich, ≥ 98%), glucose (Sigma, ≥ 99.5%), bovine serum albumin (Sigma, lyophilized powder, ≥ 96%), IgG from human serum (Sigma, reagent grade, ≥ 95%), and artificial cerebrospinal fluid (Tocris Biotechne, 99.5%).

### Synthesis of sub-20 nm plasmonic gold nanocones array (AuNCs)

The synthetic process of a sharp tip anodic aluminum oxide as a platform for AuNCs was performed based on a procedure described elsewhere. Magnetron sputtering (Q150T ES plus, Quorum) was used to achieve the desired plasmonic Au nanocones (AuNCs) structure [28]. The sputtering time (200 s) was optimized in order to achieve the best signal enhancement in combination with the available laser wavelength 785 nm.

### Apparatus

Scanning electron microscope (SEM, Scios 2 DualBeam, ThermoFisher SCIENTIFIC) was used to analyse the morphology of the sample. The high-angle annular dark-field (HAADF) image and EDS elemental mapping analysis were performed using a HRTEM Titan G2 (FEI) with image corrector on accelerating voltage 300 kV. Images were taken with BM UltraScan CCD camera (Gatan). X-ray photoelectron spectroscopy (XPS) analysis was performed on a Nexsa G2 (Thermo Fisher Scientific) with an Al Kα source (photon energy of 1486.7 eV; spot size of 100 μm). The obtained data were evaluated by using Avantage software and CasaXPS. The optical properties were investigated by reflectance measurements with a Specord250 Plus spectrometer equipped with an integrating sphere (Analytik Jena GmbH, Germany) in the 300–1100 nm range.

### Raman and SERS measurements

For the Raman measurements, Raman microscope (Thermo Scientific, USA) equipped with laser.

785 nm and software Omnic 8 was used. Presented spectra were subjected to baselines created with the asymmetric least squares smoothing mode (ALS). Stokes Raman spectra were collected in the range from 400 to 1700 cm^− 1^ with spectral resolution 1.9 cm^− 1^, achieved by implementing x10 magnification and 50 μm slit. The laser power on the sample was set to 30 mW, exposing time was set to 1 s with 20 expositions per spectrum. Spectra were typically measured in at least three independent experiments, with minimum of six replicas each.

Raman measurements of powders and stock solutions were gathered as reference spectra for SERS peak position assignment and later for the calculation of the analytical enhancement factor for the AuNCs substrate, based on the equation $\:EF=({I}_{SERS}/{c}_{SERS})\bullet\:({c}_{RamanSignal}/{I}_{RamanSignal})$, where *I* represents spectral intensity at chosen peak position, and *c* represents the concentration of the analyte. For the SERS experiments, analytes were drop-casted onto the substrates, or a small piece (around 20 mm^2^) of the substrate was incubated for 1 h in 100 µL of analyte solution. During incubation, samples were kept in the dark. All measurements were performed on a dried substrate to eliminate the influence of laser light refraction in liquid.

Raman mapping was performed with the aim of showing homogeneity of the synthesized substrate. The system was automatically re-focused before measuring each data point. The map was first normalized, and it is presented as the intensity distribution of peak height of spectral band at 678 cm^− 1^, characteristic for sertraline.

### Discrimination analysis

The discrimination analysis between serotonin and sertraline was performed in R studio (version 2023.09.1.) using R packages Chemospec [33] and Irlba [34]. First, the spectral background was removed using ALS function. Next, spectra were normalized using the partial quotient normalization (PQN) method and data were smoothed using the Savitsky-Golay approach (window 7, without derivatization). Pre-treated spectra were subjected to a partial least square discriminant analysis (further labelled as PLS-DA).

## Results and discussion

**Fig. 1:**
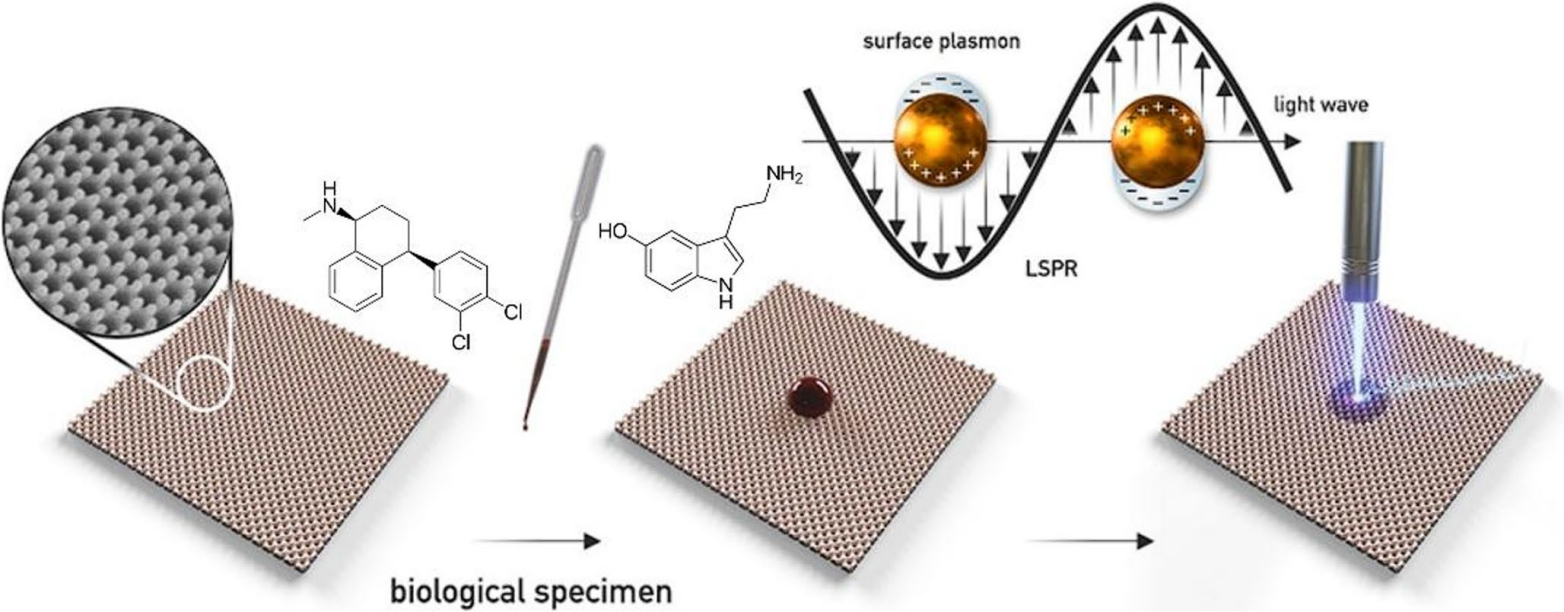
Schematic representation of the process of SERS detection of sertraline and serotonin via gold nanocone array

The worldwide increasing variety of antidepressants prompts a demand for new, more accurate and sensitive approaches allowing their analysis. Moreover, the healthcare strategy is leading towards a personalized medicine, where precise measurements of drug levels are essential for the careful tuning of therapeutic outcomes. New technologies comprised of advanced nanomaterials could provide increased specificity and sensitivity, thus push towards more reliable analytical methods allowing earlier patient recovery and safety at the same time. In this work, a novel approach for the sensing of antidepressant sertraline and neurotransmitter serotonin using surface enhanced Raman spectroscopy is presented, as depicted in Fig. 1. We applied a large-scale gold nanocone array as a reliable SERS substrate used for their detection from a solution. Their simultaneous detection from cerebrospinal fluid could be an effective perspective for the therapeutic drug monitoring of sertraline.

### Characterization of AAO-AuNCs substrate

The total process for producing a plasmonic AuNCs array is depicted in Fig. S1. To obtain the sharp tips on the AAO substrate, Al foil has undergone multiple steps of anodization and pore-widening consequently. By applying the sputtering process, the gold layer was slowly deposited and fully covered both the sharp tips and the wall of the AAO template, which generated plasmonic NCs and junctions at adjacent AuNCs.

**Fig. 2:**
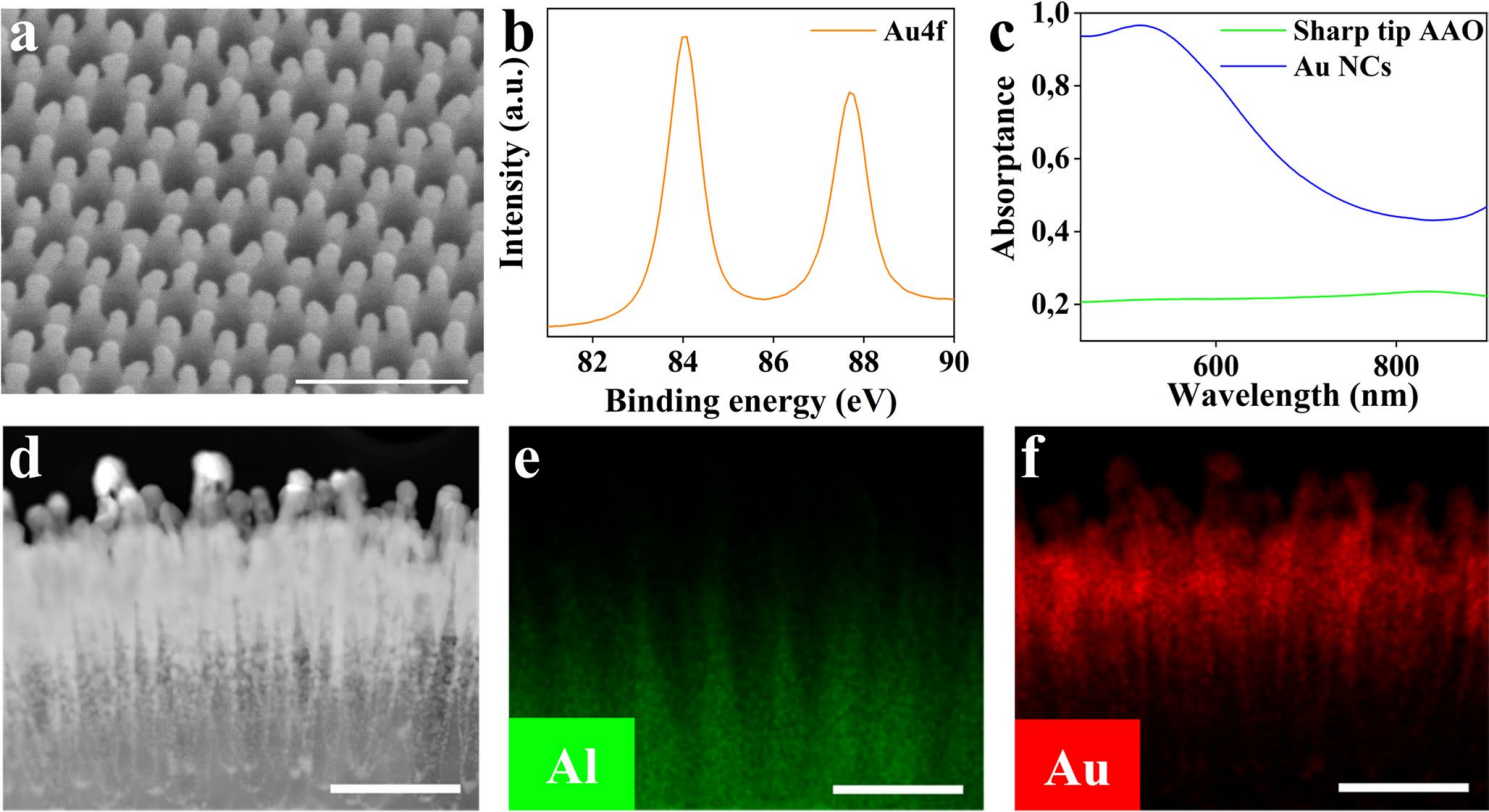
**a** The SEM image at the tilted view for AAO-AuNCs after 200 s gold sputtering. The scale bar is 200 nm. **b** the XPS spectrum of Au4f with two peaks corresponding to metallic gold. **c** the UV absorptance spectra of plasmonic AuNCs and bare sharp tip AAO. **d** HAADF images of plasmonic AuNCs with the element mapping of Al and Au **e**,** f**

SEM images display the sharp tips of the bare AAO substrate and after their subsequent coating with gold using the magnetron sputtering (hereafter AuNCs) (Fig. 2a, Fig. S2a-c). The morphological features of these nanostructures were in details characterized by FE-SEM and HAADF methods. The sharp tips were regularly formed on the AAO substrate surface, creating a hexagonally close-packed array aligned with each nanopore (Fig. S2a, b). Top-view SEM images revealed that the diameter and nanogap between adjacent sharp tips were approximately 17 nm and 42 nm, respectively, while the nanopore diameter measured around 98 nm, with an inter-pore wall thickness of about 13 nm, all in accordance with the previously published protocol [28]. The formation of sharp tips required careful optimization of anodization and pore-widening durations to ensure stable tip geometry and suitable height for subsequent metallic deposition. Following the deposition of a gold layer onto the sharp-tip AAO substrate, the Au coating uniformly covered both the tip surfaces and the inner nanopore walls, resulting in vertically aligned plasmonic Au nanocones (AuNCs) with well-defined metallic nanogaps between adjacent structures. In the top-view SEM image (Fig. S2c), AuNCs with an average size of approximately 42 nm are seen to be arranged according to the morphology of the sharp tips on the AAO substrate, indicating that the tip structure was preserved during the sputtering process. Fig. S2e confirms the high uniformity and reproducibility of AuNCs in a large-scale fabrication. The average nanogap distance between AuNCs was estimated to be approximately 14 nm (Fig. S2f), forming plasmonic hot spots capable of significantly enhancing the Raman signal of target analytes. A Raman map of a large area (414 × 368 μm) further supports the claim of uniformity through spectral features of the AuNCs substrate (Fig. S3a-c). The map was re-measured after 1 week to evaluate the stability of the substrate, and the obtained data show consistent uniformity (Fig. S3d-f). The high regularity of the AuNC array, with uniform nanogaps extending over centimeter-scale areas, was achieved through a cost-effective fabrication approach. This method therefore offers a scalable and practical alternative to conventional lithography for producing ordered plasmonic nanostructures for SERS applications.

High-angle annular dark field (HAADF) imaging combined with EDS elemental mapping was used to visualize the nanoscale structure of the AuNCs (Fig. 2d), revealing the elemental distribution of Al and Au (Fig. 2e, f, distribution of O can be found in Fig. S2d). The analysis visualized both the sputtered gold layer and the underlying sharp-tip structure on the AAO substrate, showing a clear contrast between the bright (Au-rich) and dark (oxide) regions. The oxide layer exhibited a conical nanopore morphology, with the pore diameter gradually decreasing from top to bottom. The effect of sputtering duration was addressed in our previous study [28]. In this study, the Au deposition time was fixed at 200 s, as this yielded the best plasmonic properties usable in SERS. To investigate the chemical state of the AuNCs substrate after sputtering, X-ray photoelectron spectroscopy (XPS) was performed. Figure 2b presents the Au 4f spectrum of the fabricated plasmonic AuNCs, while the corresponding Al 2p and O 1 s spectra are shown in Fig. S4. The Au 4f signal was deconvoluted into two well-resolved peaks at binding energies of approximately 84.1 eV and 87.7 eV, corresponding to Au 4f₇⁄₂ and Au 4f₅⁄₂, respectively—characteristic of metallic gold [35]. The XPS spectrum of the pristine sharp-tip AAO substrate is shown in Fig. S5, with the corresponding flat Au 4f region displayed in Fig. S5a. To evaluate the optical changes induced by Au sputtering, UV–Vis absorptance measurements were performed on the substrates before and after gold deposition (Fig. 2c). The results revealed a distinct surface plasmon resonance (SPR) peak centered at ~ 520 nm for the AuNCs substrate, confirming its plasmonic character. In contrast, the pristine AAO substrate exhibited no notable absorption features in the 400–900 nm range.

### Validation of the SERS activity of gold nanocones

**Fig. 3:**
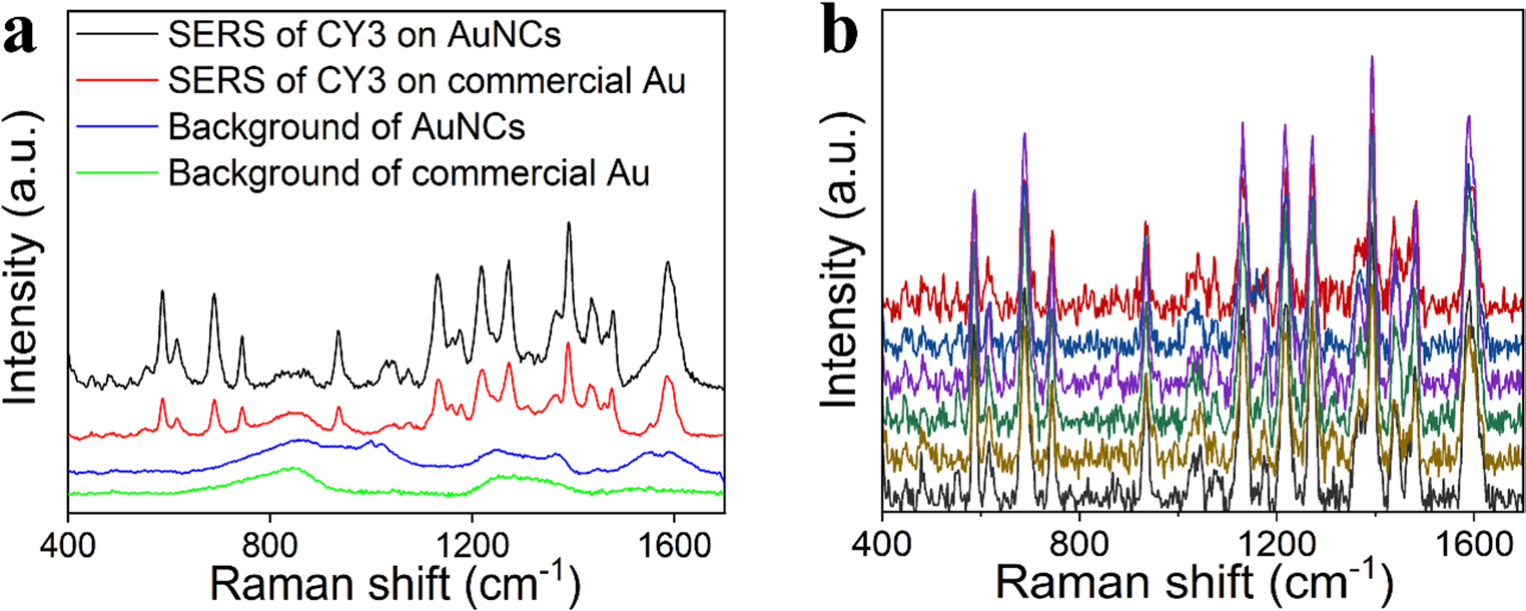
**a** SERS spectra of CY3 on AuNCs and standard commercial Au wafer at concentration 1 µg/mL, **b** Reproducibility of CY3 on AuNCs (1 µg/mL)

The SERS performance of plasmonic AuNCs substrate was evaluated by measuring a probe molecule: cyanine-3 (CY3), absorbance maximum at 540 nm [36]. Far from the excitation laser wavelength 785 nm, the enhancement of Raman signals was not expected to stem from resonance Raman. Two microlitres of CY3 were drop-casted onto the substrate, air dried, and measured. Figure 3a depicts the superior performance of the SERS intensity of plasmonic AuNCs compared to commercial gold wafer at concentration 1 µg/mL. Figure 3b shows reproducibility of the SERS signal on AuNCs substrate, and the detection at low concentration (0.1 µg/mL) can be found in the supporting information (Fig. S6a). Despite the non-resonance between laser wavelength and the SPR mode of AuNCs substrate or absorbance peak position of CY3, observation of the SERS signal could be explained by the high density of “hot spots” generating strong electromagnetic field enhancement from the sharp AuNCs and nanogap distance around 14 nm between adjacent AuNCs. Similar hypotheses were formulated in previous research [37, 38].

AuNCs stability after 1 week was tested by repeating the analysis of CY3, and the spectra can be found in Fig. 6b. There is no visible loss of analytical signal after one week, and the relative standard deviation of the intensity of spectral peak at position 585 cm^− 1^ is 6.65%.

### Serotonin and sertraline detection

Next, the duo of depression-related molecules, namely the antidepressant drug sertraline and the neurotransmitter serotonin, were detected using plasmonic AuNCs. Recently, plasma levels of another monoamine neurotransmitter, dopamine, were analysed with SERS and correlated to patients with major depression, indicating it is a topic of interest nowadays [13]. As part of the optimization process, the SERS intensity of sertraline (5 µg/mL) was clarified by comparing different volumes of drops and various incubation times. Incubating a small piece (around 20 mm^2^) of plasmonic AuNCs substrate in the analyte solution for 1-hour results in the highest intensity and better clarity for multiple peaks of sertraline across all experimental conditions (Fig. S7). Even after 1-hour long incubation, high SERS intensity indicated no degradation of the plasmonic AuNCs substrate. In conclusion, such optimal conditions were used in all following experiments for the detection of analytes in lower concentrations. Although here presented sample deposition method prolongs the measurement run-time, there is no further sample pretreatment necessary.

**Fig. 4:**
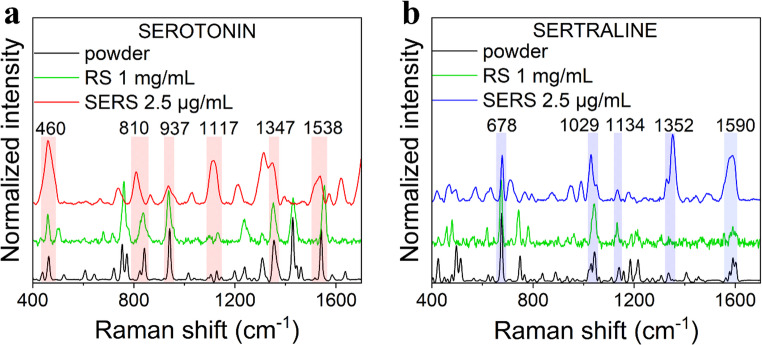
Comparison of powder spectrum with Raman and SERS signal for **a** serotonin and **b** sertraline based on plasmonic AuNCs. The spectra were normalized for better visualization of individual peak positions

Figure 4 shows powder spectra of serotonin and sertraline compared to their respective Raman spectra (RS) (1 mg/mL) and SERS spectra on AuNCs (2.5 µg/mL). This extensive comparison allows reliable peaks assignment of serotonin SERS peaks at 460, 810, 937, 1117, 1347, and 1538 cm^− 1^, and for sertraline SERS peaks at 678, 1029, 1134, 1352, and 1590 cm^− 1^. The commercial substrate exhibited barely any signal for SE or SRT at the same concentration 2.5 µg/mL (Fig. S8). By comparing the SERS signal with the spectra of the bulk powdered sample, it is possible to hypothesize that molecules of serotonin interact with the AuNC surface mostly through the indole core and hydroxy groups (found at spectral positions 460 and 1117 cm^− 1^), whereas sertraline is adsorbed through its secondary amine group (present at 1352 cm^− 1^).

Serotonin peak at 460 cm^− 1^ was previously assigned to rocking of the indole ring, OH group and H-C-H rocking, peak at 810 cm^− 1^ to C-N stretching, peak at 937 cm^− 1^ to out of phase breathing, 1347 cm^− 1^ to C = O in-plane bending, and peak at 1538 cm^− 1^ to the indole ring stretching vibration [39]. Sertraline powder peaks were reported and assigned for FT-Raman, namely at 678 cm^− 1^ for N-H twisting, 1029 cm^− 1^ for C-N stretching, 1352 cm^− 1^ for C-NH_2_, and 1590 cm^− 1^ for aromatic ring stretching [40].

**Fig. 5:**
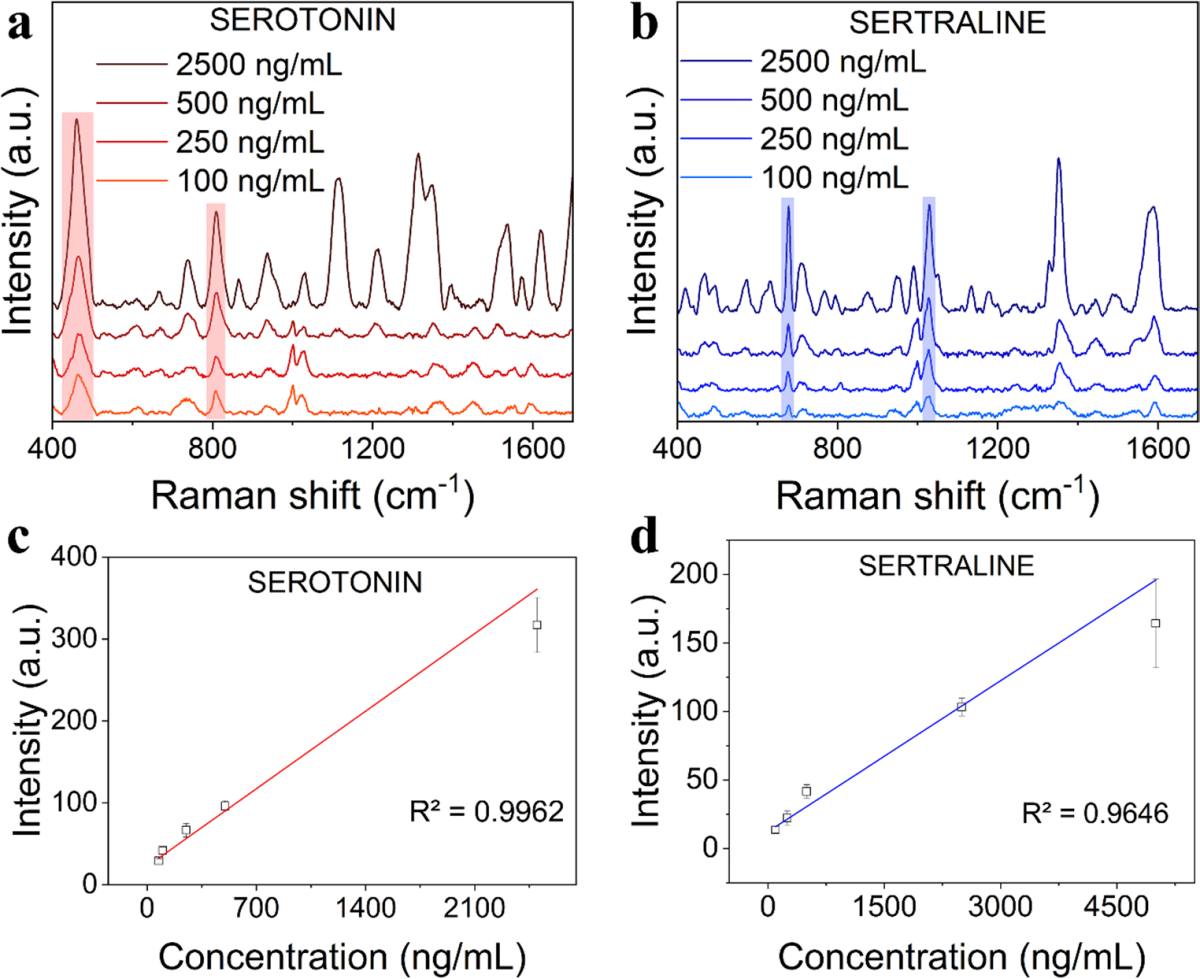
**a** SERS intensity dependence on serotonin and **b** sertraline concentrations. The marked peak positions for serotonin are at 460 and 810 cm^−1^, for sertraline at 678 and 1029 cm^−1^. **c** Linear calibration curves with R^2^ fit 0.9962 for serotonin peak 460 cm^−1^ and **d** 0.9646 for sertraline peak 678 cm^−1^. Each data point is depicted as the average value with the standard deviation of 10 individual measurements

Figure 5 shows a strong correlation between SERS intensity and the concentration of the analyte. This experiment was performed by incubating the SERS substrate in a solution containing the analyte at a defined concentration level. The linear calibration curves were assembled from five concentration points, achieving R^2^ fit of 0.9962 for serotonin peak at 460 cm^− 1^, and 0.9676 for sertraline peak at 678 cm^− 1^ (Fig. 5c, d). These specific peak positions were chosen based on their consistency within repeatability in intensity and shape. The relative standard deviation (RSD) of the substrate was again determined for sertraline peak 678 cm^− 1^ (500 ng/mL), resulting in RSD 7.15% withing one substrate, and 13.19% among three individual batches of substrates, marking AuNCs notable homogeneity and reproducibility. The data and individual spectra can be found in Fig. S9. Finally, the limit of detection (LOD) was calculated, reaching 27 ng/mL for SE and 88 ng/mL for SRT, calculated as 3σ/k ($\:\sigma\:$ is the standard deviation of the blank sample at the peak position, and k is the slope of the linear calibration curve), and the enhancement factor was 2.7·10^4^ for serotonin and 3.6·10^3^ for sertraline. The achieved LODs are comparable to other AAO/nanogap SERS substrates applied for the detection of biomedically relevant analytes [30, 41, 42]. Notably, the LOD for serotonin using plasmonic AuNCs would be applicable for serotonin screening from serum for early detection of colon cancer [19]. The SERS limit of detection for sertraline presented in this work is the lowest reported.

### Simultaneous detection

**Fig. 6:**
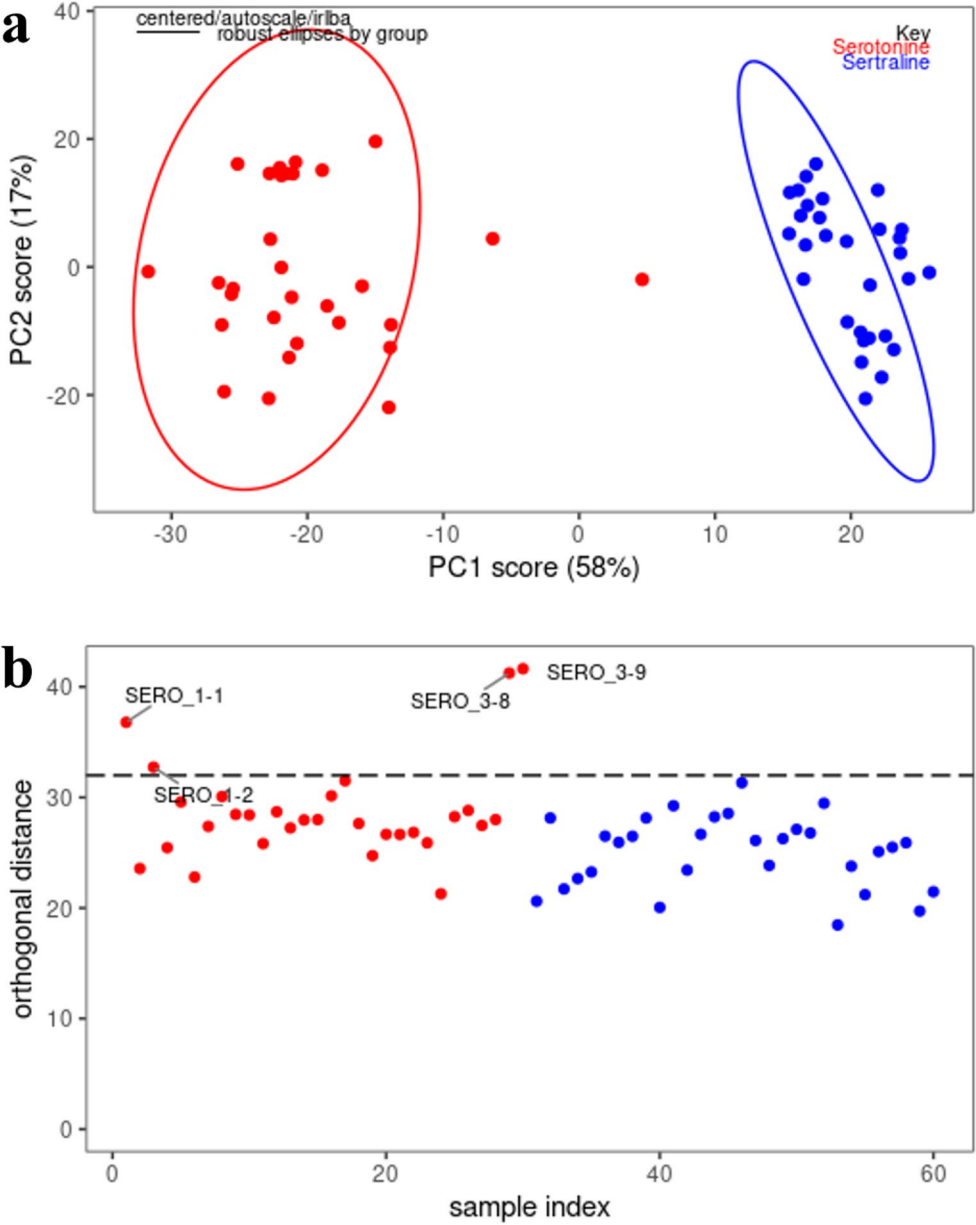
**a** Discrimination analysis of serotonin (red) and sertraline (blue) using PLS-DA method. The image shows PC1 vs. PC2; **b** analysis of orthogonal distance in the groups of serotonin (red) and sertraline (blue)

Sertraline has a direct effect on serotonin concentration in the synaptic cleft, which is mirrored in the cerebrospinal liquid. To observe patient’s response to sertraline treatment, their multiplex quantitative determination is demanded. For this purpose, a discrimination method able to distinguish a mixture of both molecules, serotonin and sertraline, has been developed. Sixty SERS spectra were analysed, namely 30 for serotonin and 30 for sertraline, with a discrimination method based on partial least square-discriminant analysis (PLS-DA) developed in R using packages described in Section 2.5. The parameters of PLS-DA were selected based on eigenanalysis, and 8 components were selected for the construction of the PLS model. Results are shown in Fig. 6. The method can discriminate between serotonin and sertraline in a statistical space defined by principal component 1 (PC1) (explaining 58% of variance) and principal component 2 (PC2) (explaining 17% of variance), as shown in Fig. 6a. Figure 6b shows orthogonal distance for all 60 analysed samples, which indicates a reasonable level of variation between sample present within one group.

The samples containing serotonin or sertraline (*n* = 30 and 30, concentration = 500 ng/mL) were randomized and divided into training (*n* = 45) and testing (*n* = 15) groups. The resulting model shown accuracy of the PLS-DA detection 0.933 (*p* < 0.012), measured on a testing group consisting of randomly selected samples. The corresponding sensitivity is 0.87 and specificity 0.99.

**Fig. 7:**
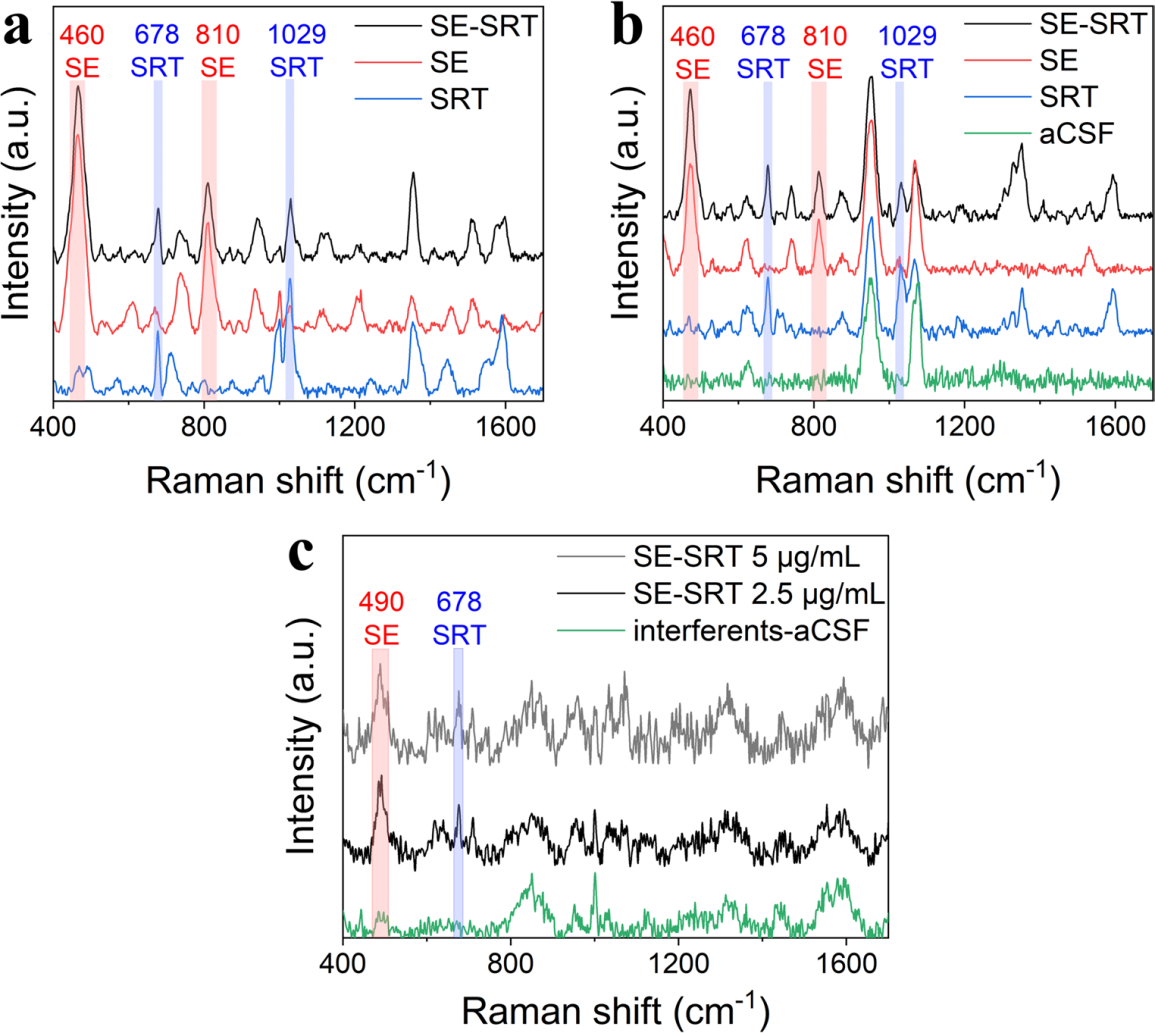
**a** SERS spectra of an equimolar mixture of serotonin-sertraline (SE-SRT) compared to their individual SERS spectra at concentration 500 ng/mL in MQ water, **b** 2.5 µg/mL in artificial cerebrospinal fluid (aCSF), and **c** 2.5 µg/mL in interferents-aCSF. Peaks at 460 and 810 cm^− 1^ are assigned to serotonin, and peaks at 678 and 1029 cm^− 1^ to sertraline. The serotonin peak is shifted in **c** to 490 cm^− 1^ because of its interaction with biomolecules

Furthermore, simultaneous detection of serotonin and sertraline from one sample was achieved from both MQ water and artificial cerebrospinal fluid (aCSF). ACSF was chosen as a matrix for its clinical relevance to the simultaneous determination of serotonin and sertraline levels [6]. Figure 7 shows the comparison of individual spectra of serotonin, sertraline, and their equimolar mixture in (a) MQ water at concentration 500 ng/mL and (b) aCSF at concentration 2.5 µg/mL. Despite some peak positions emerging from blank aCSF solution, they do not overlap with either of the most prominent analyte peaks – 460 and 810 cm^− 1^ that are attributed to serotonin, and 678 and 1029 cm^− 1^ attributed to sertraline. Moreover, the intensity of these peaks is almost identical in individual spectra and the mixture in both environments, further proving reliability and replicability of the plasmonic AuNCs substrate, a necessity for potential clinical application. There are some overlapping peaks between the analytes, which can be explained by their structural similarities such are the secondary amine and an aromatic core. For this reason, great care was put into choosing the characteristic peaks for quantification with no considerable overlap, where namely band at 460 cm^− 1^ was selected for SE, and the spectral band at 678 cm^− 1^ for SRT. The detected concentration of serotonin from aCSF is lower than what was reported by *Moody et al.* using a Au colloid and the same laser wavelength 785 nm [43], which again indicates Au nanocone arrays to be superior SERS substrates to Au colloids.

Finally, to tests whether larger biomolecules naturally present in the cerebrospinal fluid might hinder SERS signal of targeted analytes, an interference study was performed. Albumin, human IgG, and glucose, as the most abundant molecules in CSF, were dissolved in aCSF in physiological concentration levels – 0.19 mg/mL, 0.023 mg/mL, and 0.6 mg/mL, respectively [44]. The resulting spectra of a SE-SRT mixture (c = 2.5 µg/mL) can be seen in Fig. 7c. Although the interferents partially blocked the substrate, signal of both serotonin and sertraline was clearly detected. The higher noise in the spectra and lower intensities of peaks are attributed to the complexity of the matrix. The peak of serotonin was shifted to 490 cm^− 1^, which can be explained by the interaction of its main structural feature – indole core – with large biomolecules. To confirm the origin of signal from serotonin, higher concentration (5 µg/mL) was also measured, and the peak rose by 16%. he LODs were calculated as the S/N (3:1) ratio, reaching 1.5 µg/mL for serotonin and 1.2 µg/mL for sertraline. This LOD of SRT still corresponds to sub-physiological values in CSF [45], further supporting the usability of AuNCs substrate as a reliable platform for therapeutic drug monitoring.

## Conclusion

In this work, highly reproducible and sensitive SERS detection of neurotransmitter serotonin and SSRI drug sertraline was achieved, reaching limits of detection 27 and 88 ng/mL, respectively. The analytical enhancement factor of the AuNCs substrate is 10^4^ for serotonin and 10^3^ for sertraline. The SERS sensitivity for these target molecules is due to the high density of hot spots generated by the sharp AuNCs and sub-20 nm gap distance between adjacent AuNCs. The presented simultaneous SERS detection of serotonin and sertraline from artificial cerebrospinal fluid containing interferents suggests future use of the developed spectroscopic approach for point-of-care monitoring of sertraline medication effectivity in terms of therapeutic drug monitoring (TDM). Importantly, this sensitive approach has a high potential for a fast and reliable TDM from clinical samples expanding beyond cerebrospinal fluid, as well as detecting e.g. drug contaminants from wastewater.

## Supplementary Information

Below is the link to the electronic supplementary material.

Supplementary Material 1 (DOCX 3.79 MB)

## Data Availability

Data are available on Zenodo repository : https://zenodo.org/records/18284194.
