# Supplementary material for: Precise and simultaneous SERS detection of sertraline and serotonin on large-scale sub-20 nm plasmonic gold nanocone arrays
Source: Mikrochim Acta. 2026 Jun 6;193(7):450. doi: 10.1007/s00604-026-08164-7 (PMC13241410; doi:10.1007/s00604-026-08164-7)
Supplement: Supplementary file 1 — Supplementary Material 1 (DOCX 3.79 MB) [file 604_2026_8164_MOESM1_ESM.docx]

Supporting information

**Precise and Simultaneous SERS Detection of Sertraline
and Serotonin on Large-Scale Sub-20 nm Plasmonic
Gold Nanocone Arrays**

Klára Gajdošová^a,b^, Thanh-Lam Bui^a,c^, Zuzana Chaloupková^a^, S. M. Hossein Hejazi^a,c^, Radek Zbořil^a,c^, Štěpán Kment^a,c,*^, Václav Ranc^a,d,*^, Kateřina Poláková^a,*^

*^a^ Regional Centre of Advanced Technologies and Materials, Czech Advanced Technology and Research Institute (CATRIN), Palacký University Olomouc, Šlechtitelů 27, 783 71 Olomouc, Czech Republic*

*^b^ Department of Physical Chemistry, Faculty of Science, Palacký University Olomouc, 17 Listopadu 12, 771 46 Olomouc, Czech Republic*

*^c^ Nanotechnology Centre, Centre for Energy and Environmental Technologies, VSB – Technical University of Ostrava, 17. listopadu 15, 708 00 Ostrava-Poruba, Czech Republic*

*^d^ Institute of Molecular and Translational Medicine, Faculty of Medicine and Dentistry, Palacký University and Faculty Hospital Olomouc, Hněvotínská 5, 775 15 Olomouc, Czech Republic*

** Corresponding authors:* [*stepan.kment@upol.cz*](mailto:stepan.kment@upol.cz) *(Š. Kment),* [*vaclav.ranc@upol.cz*](mailto:vaclav.ranc@upol.cz) *(V. Ranc),* [*katerina.polakova@upol.cz*](mailto:katerina.polakova@upol.cz) *(K. Poláková)*


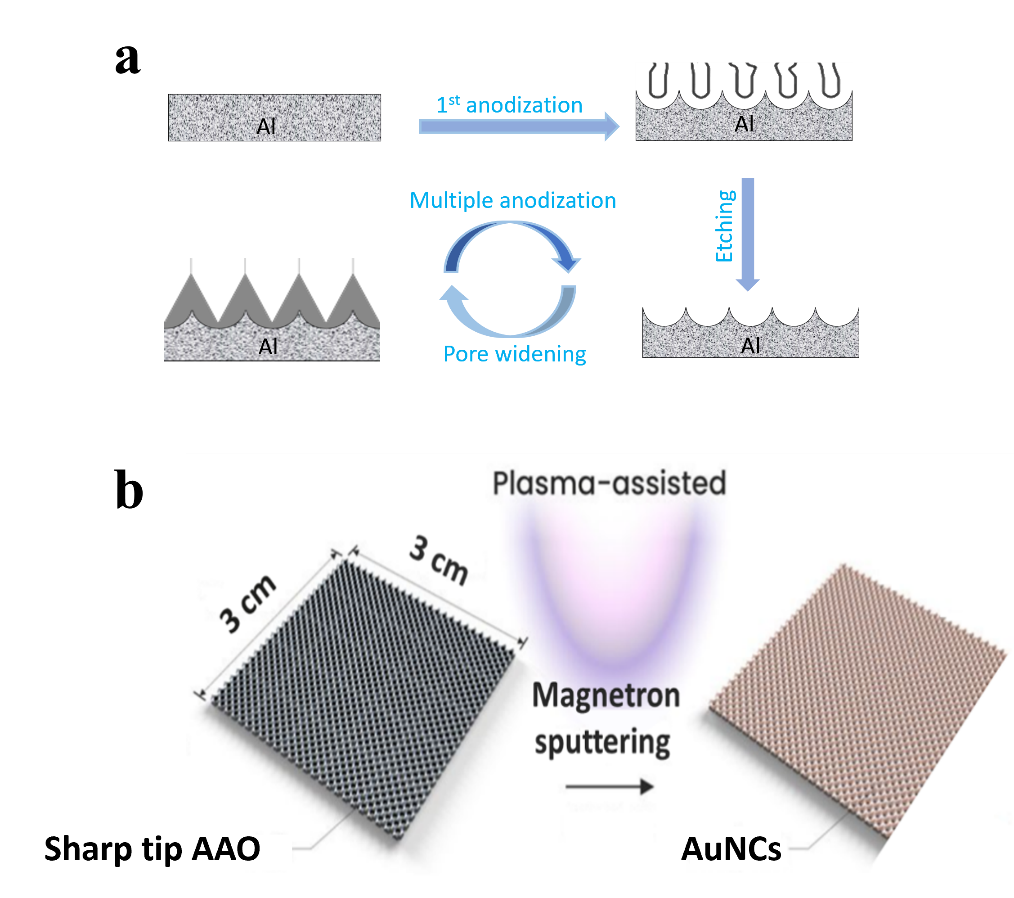


**Fig. S1:** Schematic representation of the sharp tip anodic aluminum oxide (AAO) substrate fabrication (a), depiction of gold nanocone (AuNCs) generation by the magnetron sputtering process (b).


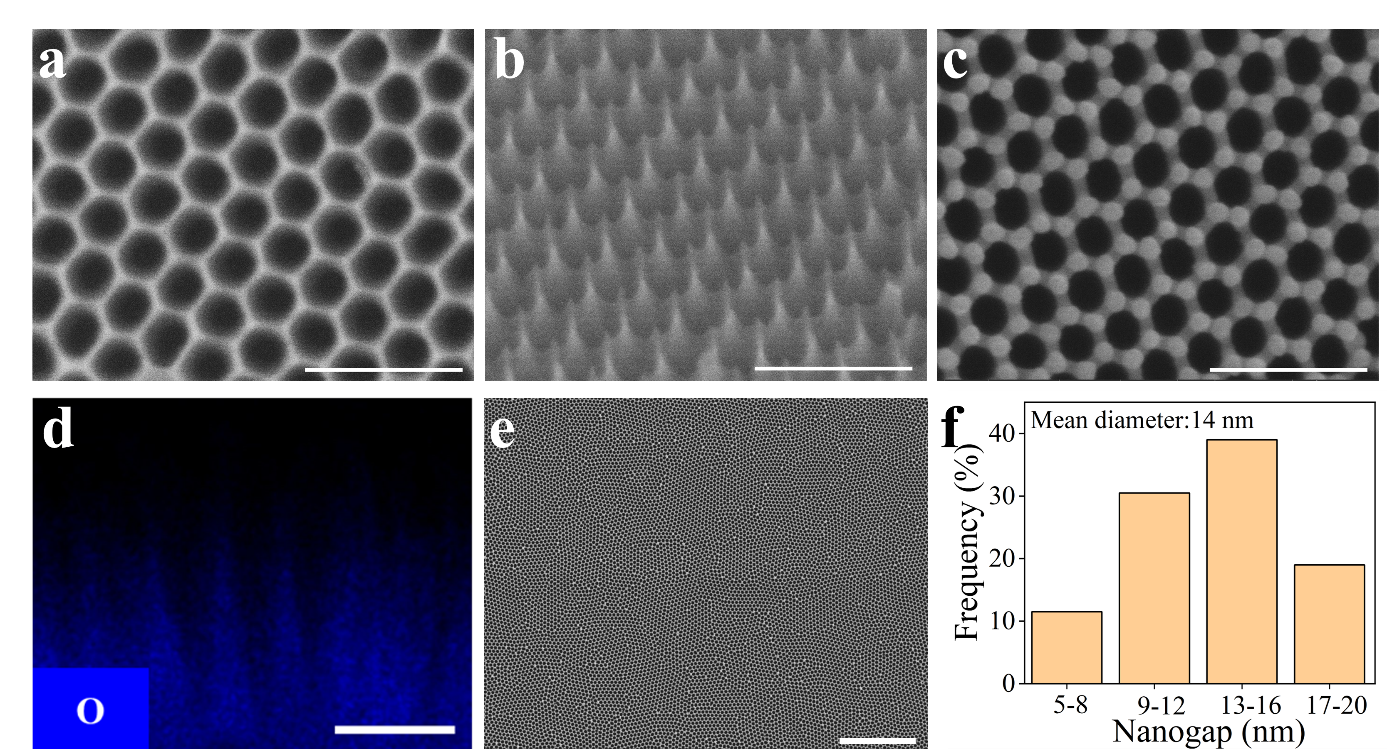


**Fig. S2:** (a, b) The SEM image at the top and tilted view for bare AAO, (c) top view of AAO-AuNCs after 200s gold sputtering. The scale bars are 200 nm. (d) HAADF image of AAO-AuNCs with elemental mapping of O. (e) The large-scale SEM image of AuNCs with scale bar 2 µm and (f) the average nanogap distance of adjacent AuNCs, based on 200 measurements.


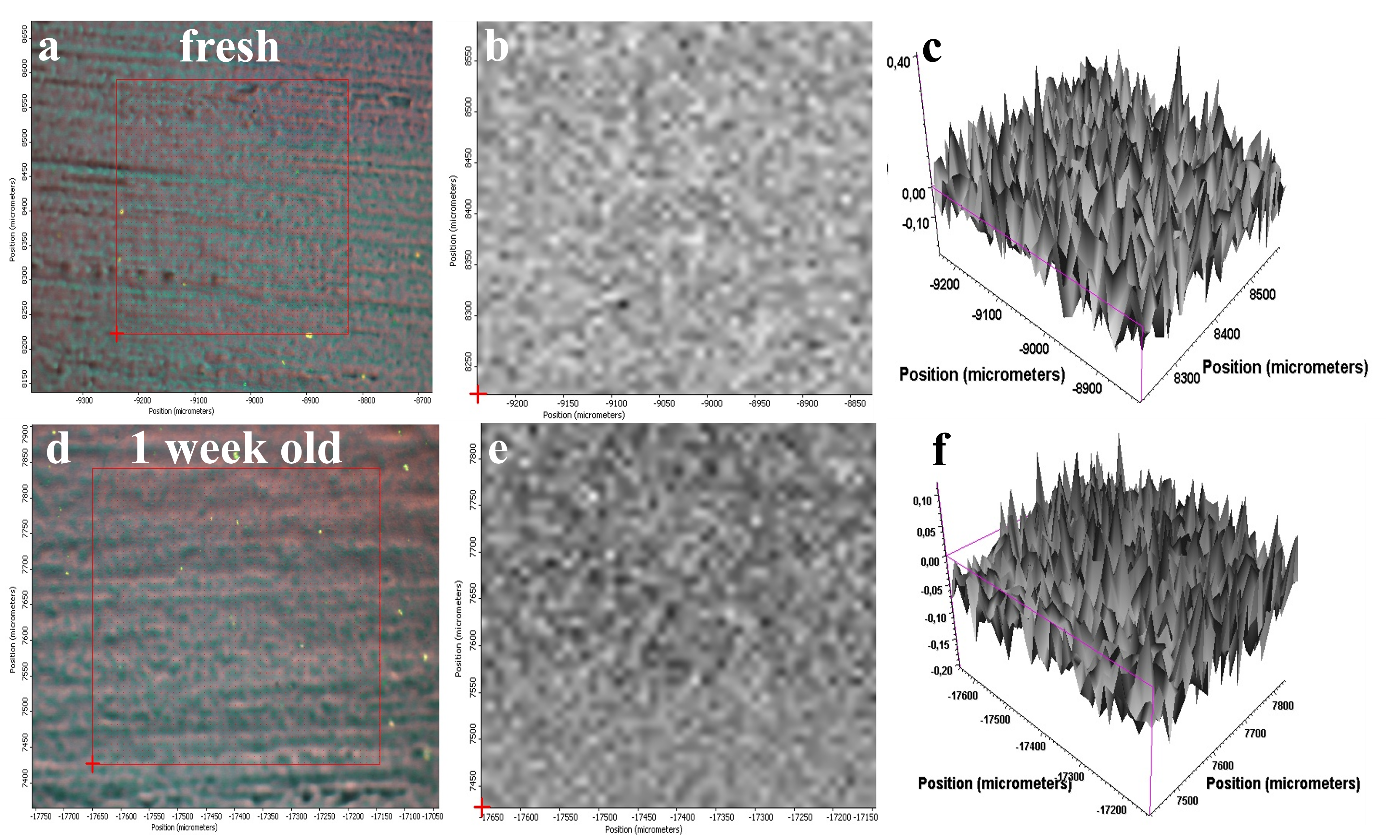


Fig. S3: Raman maps of a large area (47 x 47 points over 414 x 368 µm) of a fresh AAO-AuNCs substrate (b) and 1 week old substrate (e), correlated to their optical images (a, d) and a peak area distribution of 678 cm^-1^ (e, f) where sertraline’s, our targeted analyte’s, main peak will be present. The optical image of the processed map was evaluated using Image J. The average relative intensity after baseline subtraction, smoothing, and normalization is 165 (for fresh substrate) and 131 (for 1 week old substrate), and the RSD on this large area comprised of over 2200 points is 12.2 % for the fresh substrate, and 16.6 % for the 1 week old substrate. The decrease in intensity is 20 %.


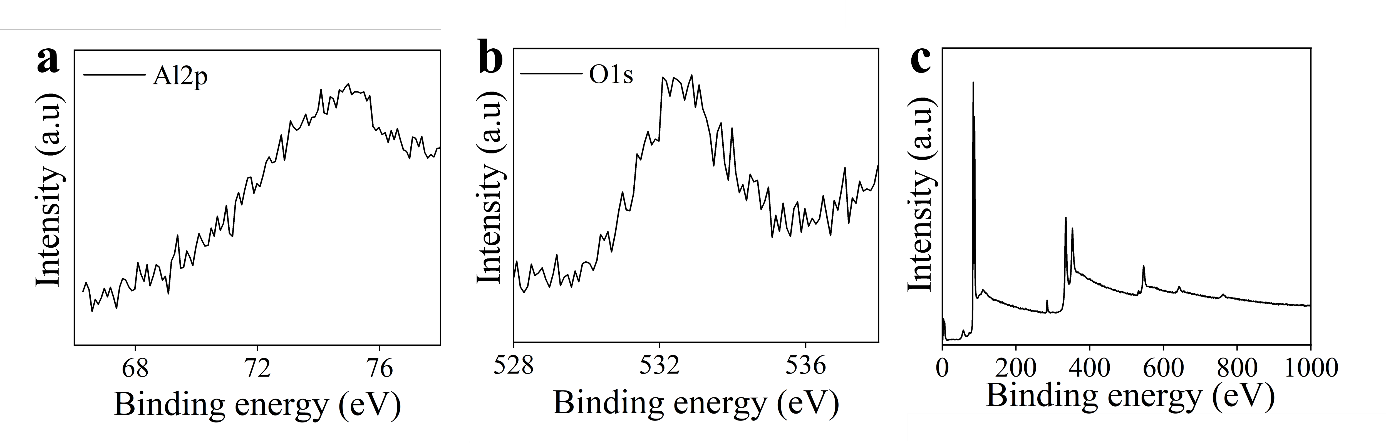


**Fig. S4**: The XPS spectra of (a) Al2p, (b) O1s, (c) full spectrum of Au NCs.


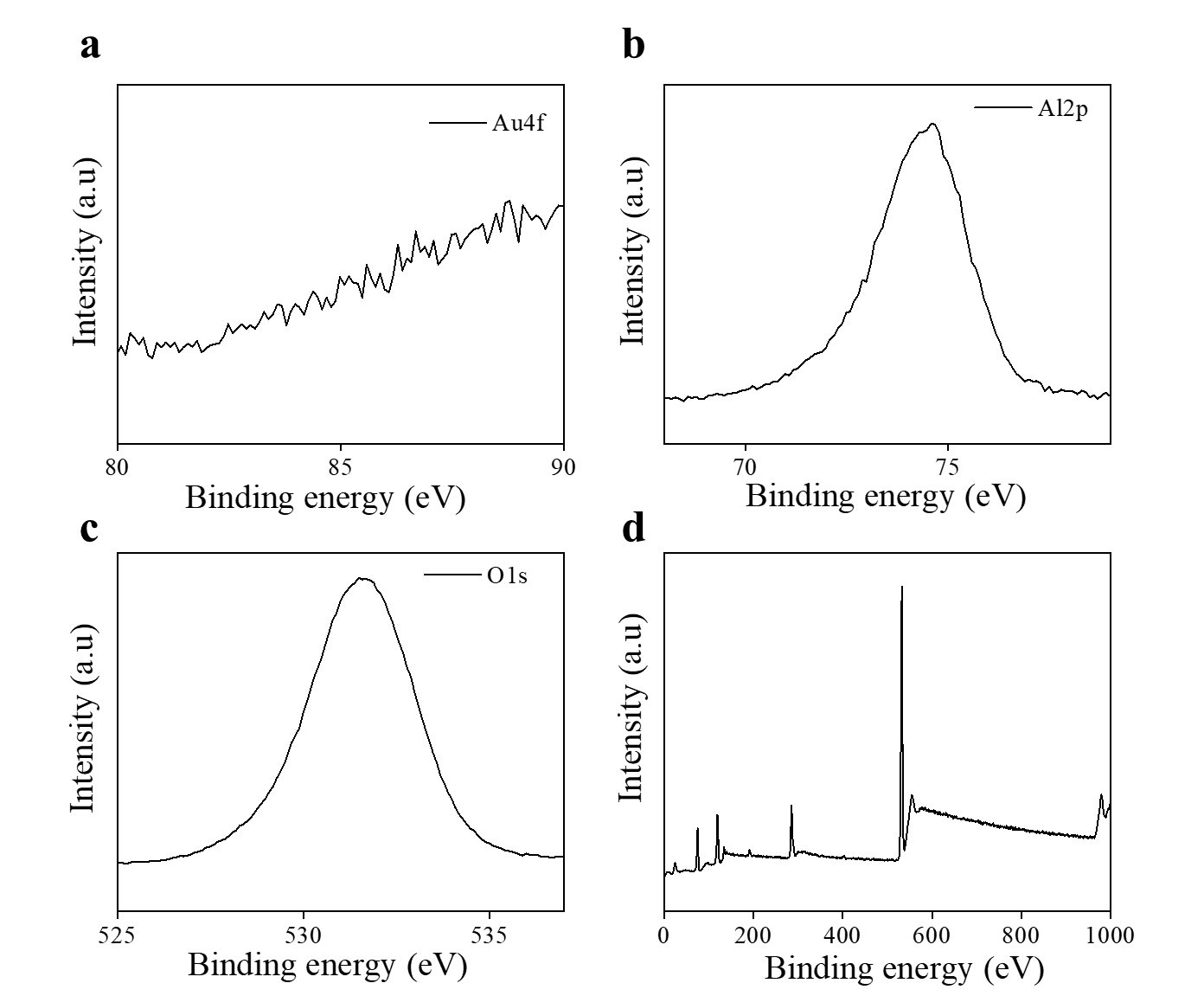


**Fig. S5**: The XPS spectra of (a) Au4f, (b) Al2p, (c) O1s and (d) full spectrum of bare sharp tip AAO substrate.


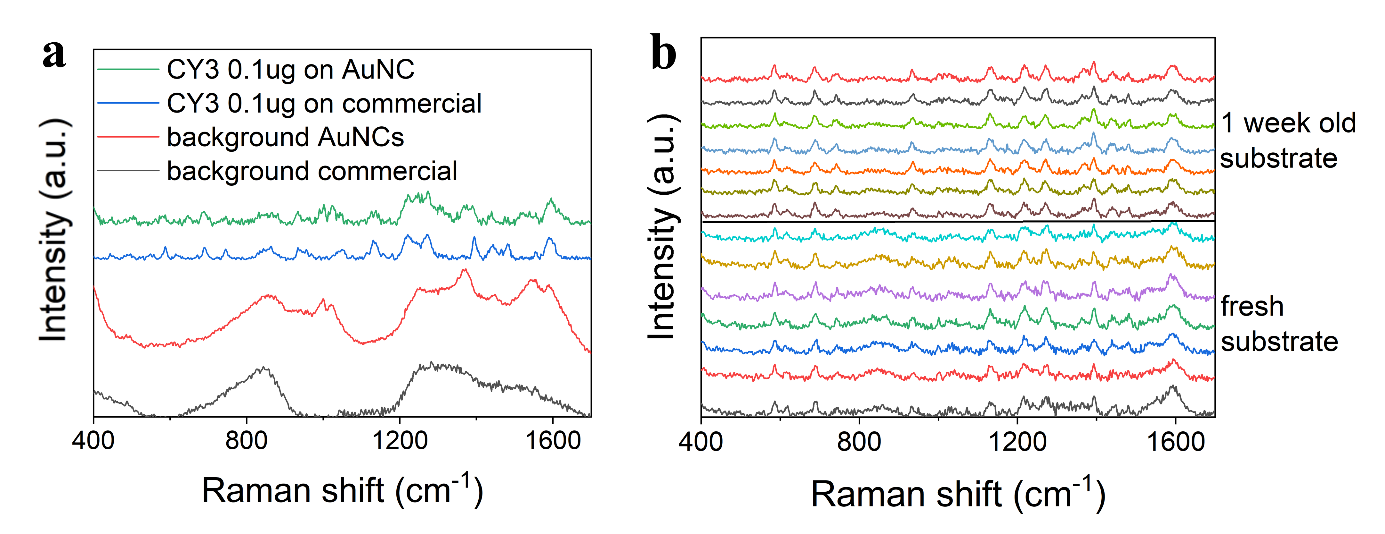


**Fig. S6**: (a) SERS signal of Raman reporter CY3 at concentration 0.1 µg/mL on AuNCs substrate and commercial Au wafer. (b) Substrate stability after 1 week, depicted as 7 spectra of Raman reporter molecule CY3 (c = 1 µg/mL) measured on a fresh substrate and 7 spectra measured on a 1 week old substrate. There is no visible loss of signal intensity. The relative standard deviation (RSD) between all 14 spectra, peak 585 cm^-1^, is 6.65 %.


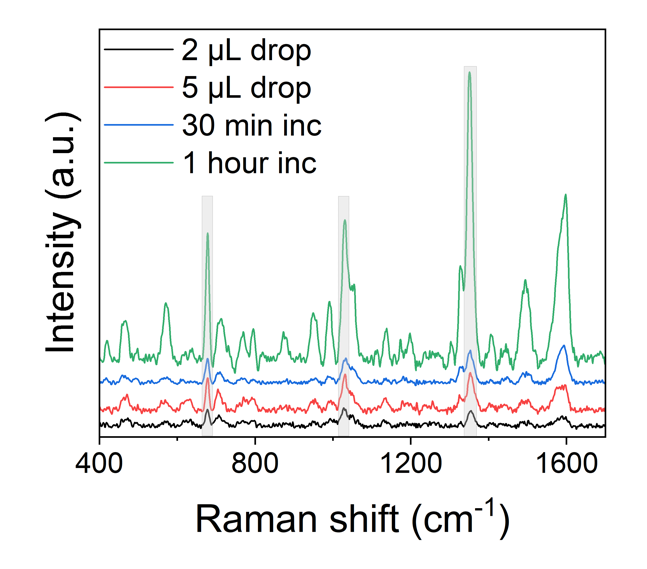


**Figure S7**: Comparison of different volumes of a drop (2 or 5 µL), and of different incubation times (30 mins or 1 hour) of the substrate in 100 µL of sertraline (5 µg/mL) on AuNCs substrate. The most prominent peaks of sertraline visible in all spectra are located at 678, 1029, and 1352 cm^-1^.


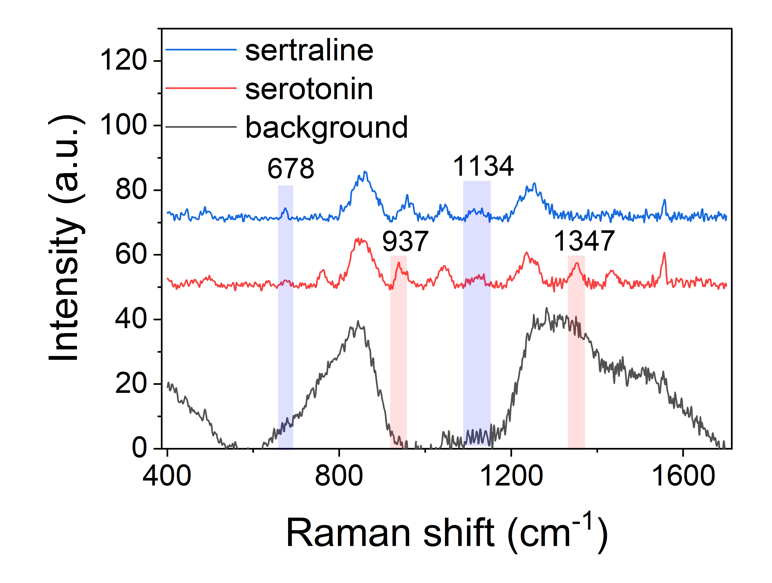


**Fig. S8**: SERS spectra of serotonin and sertraline (2.5 µg/mL) on commercial gold wafer.


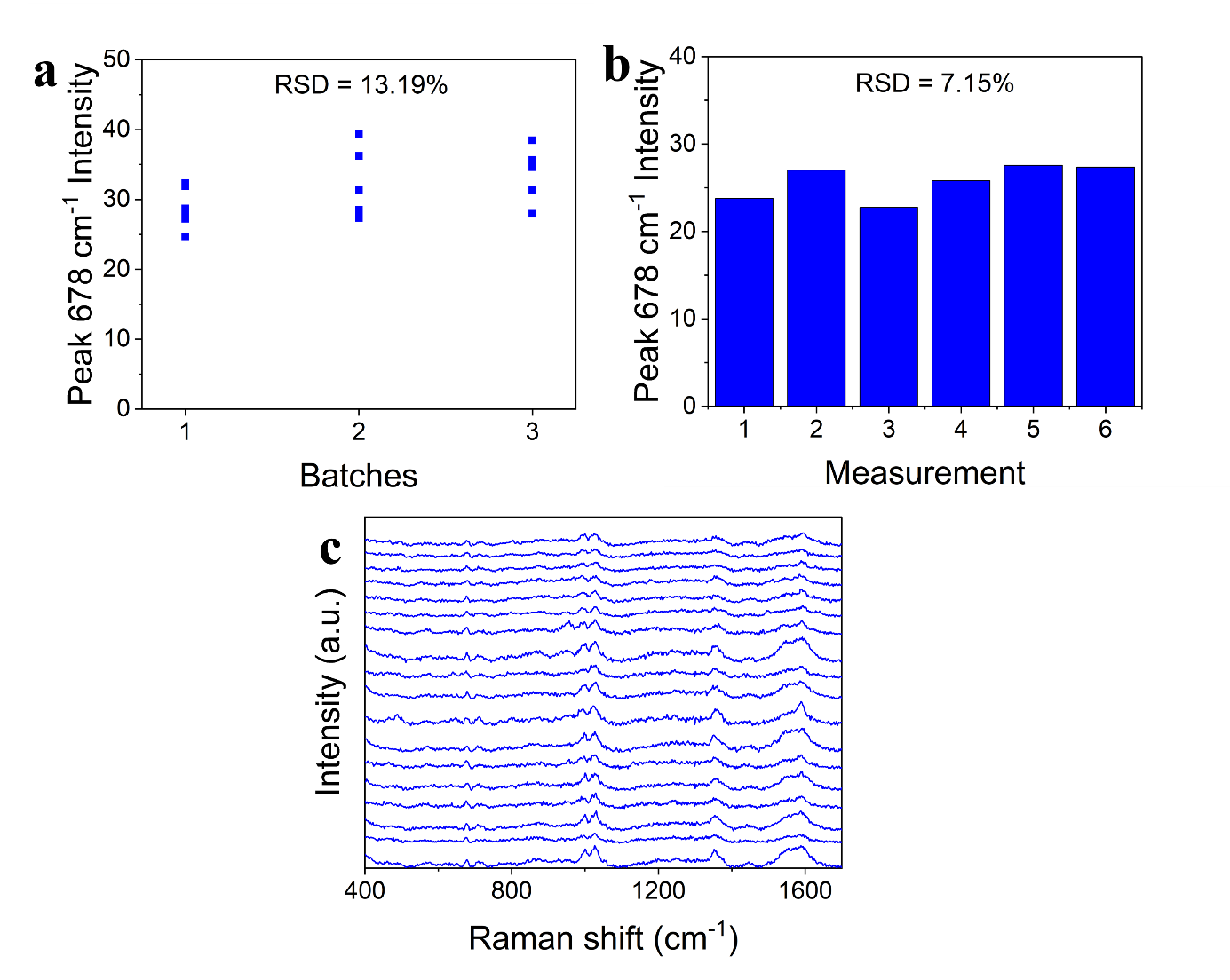


**Fig. S9**: Intensity of sertraline (500 ng/mL) peak 678 cm^-1^ on three individual batches of AuNCs substrate – six repetitions for each substrate (a) and six individual measurements within one substrate (batch 1) (b), and the individual spectra (c), allowing calculation of the relative standard deviation (RSD) between the SERS signals.
